# Gram-stain-based antimicrobial selection reduces cost and overuse compared with Japanese guidelines

**DOI:** 10.1186/s12879-015-1203-6

**Published:** 2015-10-26

**Authors:** Tomohiro Taniguchi, Sanefumi Tsuha, Soichi Shiiki, Masashi Narita

**Affiliations:** Division of Infectious Diseases, Okinawa Chubu Hospital, 281 Miyazato, Uruma, Okinawa 904-2293 Japan

**Keywords:** Gram stain, Japanese guidelines, Antimicrobial stewardship

## Abstract

**Background:**

The Gram stain has been used as an essential tool for antimicrobial stewardship in our hospital since the 1970s. The objective of this study was to clarify the difference in the targeted therapies selected based on the Gram stain and simulated empirical therapies based on the antimicrobial guidelines used in Japan.

**Methods:**

A referral-hospital-based prospective descriptive study was undertaken between May 2013 and April 2014 in Okinawa, Japan. All enrolled patients were adults who had been admitted to the Division of Infectious Diseases through the emergency room with suspected bacterial infection at one of three sites: respiratory system, urinary tract, or skin and soft tissues. The study outcomes were the types and effectiveness of the antibiotics initially selected, and their total costs.

**Results:**

Two hundred eight patients were enrolled in the study. The median age was 80 years. A significantly narrower spectrum of antibiotics was selected based on the Gram stain than was selected based on the Japanese guidelines. The treatments based on the Gram stain and on the guidelines were estimated to be equally highly effective. The total cost of antimicrobials after Gram-stain testing was less than half the cost after the guidelines were followed.

**Conclusions:**

Compared with the Japanese guidelines, the Gram stain dramatically reduced the overuse of broad-spectrum antimicrobials without affecting the effectiveness of the treatment. Drug costs were reduced by half when the Gram stain was used. The Gram stain should be included in all antimicrobial stewardship programs.

## Background

Antibiotic resistance is a serious worldwide threat to public health [1]. The inappropriate use of antimicrobials has been shown to cause the emergence and transmission of multidrug-resistant bacteria [2–5]. A number of interventions have been proposed because the cost and consequences of antibiotic resistance are enormous [6]. Several kinds of antimicrobial stewardship programs, including an interventional approach, have reduced the inappropriate use of antibiotics, with economic savings and without affecting patient mortality [3, 5–9].

In Japan, the Japanese Association for Infectious Diseases (JAID) and the Japanese Society of Chemotherapy (JSC) jointly published antimicrobial guidelines in 2012 called “The JAID/JSC Guide to Clinical Management of Infectious Diseases 2011” [10]. This was the first publication written in Japanese and edited by these two leading societies for use by all clinicians in Japan. The guide includes all major infectious diseases, and was expected to reduce the misuse of antibiotics. However, the guidelines generally recommend the use of broad-spectrum antimicrobials that treat various kinds of organisms [11].

At Okinawa Chubu Hospital, all in-house staff members, including trained resident physicians (postgraduate year 1 or 2), perform Gram stains at the bedside in the emergency room to select the appropriate antibiotic for each patient [12, 13]. We believe that targeted antimicrobial therapy based on the point-of-care Gram stain has suppressed the emergence of resistant organisms [13].

Gram-stain-based therapy has fallen from use in the rest of the world. In the United States of America, the bedside Gram stain was discontinued after the Clinical Laboratory Improvement Amendments of 1988 required that staff members be accredited to interpret Gram stains [14]. Other factors were also involved, including the outsourcing of specimens and the economic pressures to reduce costs [14]. Therefore, no-one has compared the antimicrobial treatments selected based on the traditional Gram stain with those selected based on the latest guidelines.

The overall objective of this study was to clarify the difference in the targeted therapies selected based on the Gram stain and simulated empirical therapies based on the guidelines in use in Japan. The specific objectives were to compare the types and effectiveness of the antibiotics initially selected for treatment and the total costs of these antimicrobials.

## Methods

### Study setting

This was a hospital-based, prospective descriptive study. The study setting was Okinawa Chubu Hospital, which is located in the central area of Okinawa, a subtropical region of Japan. The University of Hawaii has supported the clinical education of the staff at this hospital with a Postgraduate Medical Education Program since 1966. Approximately 39,000 patients visit the emergency department annually and nearly 14,000 patients are hospitalized each year [15]. Patients enrolled in the study attended between May 2013 and April 2014. All adult patients who were enrolled were suspected of a bacterial infection of the pulmonary system, urinary tract, or skin and soft tissue, and were newly admitted to the Division of Infectious Diseases through the emergency room. No other sites of infection were included in the study. These three infection sites constituted over 90 % of our hospitalized patients [15]. The exclusion criteria were 1) diseases that were not included in the JAID/JSC Guide, such as pulmonary abscess, empyema, renal abscess, or prostate abscess; 2) more than one simultaneous site of infection, because this situation is not covered by the guidelines; 3) a diagnosis of something other than a bacterial infection when the culture results were received, because this study was only intended to compare the three most common sites of bacterial infection; and 4) all human immunodeficiency virus (HIV)-infected patients, because their clinical courses, such as *Pneumocystis* pneumonia, are quite different from those of community-acquired pneumonia. Infections that occurred after admission, such as *Clostridium difficile*-associated infections, were not included, for simplicity.

### Data collection

All patient information was collected from medical charts, including the types and doses of the antibiotics administered, all the bacteria isolated, changes in antibiotics after culture results, and the duration of antimicrobial use during hospitalization. All point-of-care Gram stains of sputum or urine samples were performed at the bedside by in-house staff members in the emergency room. Positive blood cultures were identified from the initial two sets of blood cultures. If the detected organisms were considered skin contaminants, the samples were classified as blood-culture negative.

### Definitions

According to the JAID/JSC Guide, pulmonary infection is classified as community-acquired pneumonia or aspiration pneumonia; urinary tract infection as pyelonephritis, complicated pyelonephritis, urosepsis, prostatitis, or catheter-related pyelonephritis; skin and soft tissue infection as cellulitis, severe cellulitis, or methicillin-resistant *Staphylococcus aureus* (MRSA)-suspected cellulitis. We diagnosed urosepsis and severe cellulitis as present in patients with a systolic blood pressure of <90 mmHg upon arrival, or in patients who did not respond to the administration of intravenous fluid. Complicated pyelonephritis was considered if a patient had a neurogenic bladder, calculi, prostate hyperplasia, an anatomical defect, or diabetes mellitus, or if they took immune suppressants or were pregnant.

Penicillins and first- or second-generation cephalosporins were defined as narrow-spectrum antibiotics; piperacillin/tazobactum, fourth-generation cephalosporin, carbapenems, and vancomycin as broad-spectrum antibiotics; and all other antibiotics as intermediate-spectrum antibiotics.

### Antimicrobial selection

In our hospital, *Streptococcus pneumoniae*, *Haemophilus influenzae*, *Moraxella catarrhalis*, *Klebsiella pneumoniae*, *Pseudomonas aeruginosa*, and anaerobes were differentiated in Gram-stained sputum by physicians in the emergency room [13]. The first-choice antimicrobial was selected based on local antibiotic-resistance patterns, which are updated every year by the hospital’s Microbiology Laboratory.

In Gram-stained sputum, Gram-positive diplococci suggested *S. pneumoniae*, and ampicillin was selected [13]. Small Gram-negative coccobacilli suggested *H. influenzae*, and a third-generation cephalosporin (cefotaxime or ceftriaxone) was selected [13]. Gram-negative diplococci suggested *M. catarrhalis*, and ampicillin-sulbactam was selected [13]. Gram-negative rods suggested Enterobacteriaceae, such as *K. pneumoniae*, and a second- or third-generation cephalosporin was selected [13]. Small Gram-negative rods suggested *P. aeruginosa*, and an antipseudomonal agent, such as piperacillin, ceftazidime, imipenem, meropenem, ciprofloxacin, or tobramycin, was selected [13]. Polymicrobial flora suggested the aspiration of oral anaerobes, and ampicillin-sulbactum was selected [13].

In Gram-stained urine, Gram-negative rods suggested Enterobacteriaceae, such as *Escherichia coli* or *K. pneumoniae*, and a second-generation cephalosporin, such as cefotiam, was selected [13]. However, if a patient was at high risk of a drug-resistant organism, such as one expressing extended-spectrum beta-lactamases (ESBLs), cefmetazole or carbapenem was selected. Small Gram-negative rods suggested *P. aeruginosa*, and an antipseudomonal agent was selected [13]. Gram-positive cocci in chains suggested *Streptococcus* or *Enterococcus*, and ampicillin or vancomycin was selected.

In skin and soft tissue infections, specimens were not usually collected unless a subcutaneous abscess developed. Cefazolin, which is effective for both *Streptococcus* and *Staphylococcus*, was selected. If the risk of MRSA infection was high, clindamycin or vancomycin was added. For immunocompromised hosts, such as those with liver cirrhosis, a third-generation cephalosporin was selected as they are effective for Gram-negative organisms, such as *Vibrio* or *Aeromonas*, and Gram positive organisms.

Within these classifications, we compared two groups: one group of patients whose antibiotic was selected based on the point-of-care Gram stain in this study, and the other group with a simulated choice of antibiotic and dosage based on the Japanese guidelines.

### Outcomes

The types of antimicrobials selected based on the Gram stain were real, whereas those based on the guidelines were simulated.

The evaluation of their effectiveness was based on culture results. If the cultured pathogen was susceptible in vitro and the clinical response was also favorable, the initially chosen antibiotic was continued or its spectrum narrowed down, and it was classified as “effective”. If the cultured pathogen was resistant in vitro and the clinical response was also unfavorable, a narrow-spectrum antibiotic was changed to a broader-spectrum antibiotic, and it was classified as “ineffective”. If the cultured pathogen was resistant in vitro, but effective in vivo, it was classified as “unknown”. If the culture result showed normal flora in the sputum or was negative, the effectiveness was evaluated from the clinical course. If a narrow-spectrum antimicrobial was effective, we considered that a broader antimicrobial in the simulation would automatically be effective. If this estimation was impossible, we considered it “unknown”.

The total antibiotic cost during hospitalization was determined using the original pharmaceutical price in Japanese yen, determined by the Ministry of Health, Labour and Welfare in Japan, and the days of intravenous antibiotic use.

### Ethics

Gram-stain-based antimicrobial therapy is the standard care at our hospital. This was an observational study, so written informed patient consent was deemed unnecessary. The study proposal was approved by the Institutional Review Board of Okinawa Chubu Hospital.

### Statistical analysis

For continuous valuables, the means and standard deviations were described for normal distributions, and the medians and interquartile ranges were described for skewed distributions. The *χ*^2^ test or Fisher’s exact test was used to analyze categorical variables, and were calculated with the Stata software (version 12.1; StataCorp, College Station, TX, USA).

## Results

Two hundred eight patients were enrolled in the study. The median age was 80 years, and the interquartile range was 64–87 years. Table 1 shows the basic characteristics of the patients. Performance status was classified according to the Eastern Cooperative Oncology Group scale [16]. Of the infection sites, the urinary tract was the most commonly affected, and skin and soft tissue was the second most commonly affected. One patient who contracted a pulmonary infection in another hospital and was transferred to our hospital was included in the aspiration pneumonia group because the patient was bedridden. Twenty-nine patients had been treated with antibiotics within the previous 48 h, so bacterial cultures were likely to be negative in many of them.

**Table 1:** Basic patient characteristics

	*N* = 208
Age (median, interquartile range)	80 (67–87)
Male	71 (34.1 %)
Performance status	
0–2	126 (60.5 %)
3 (in bed or chair more than 50 %)	41 (19.7 %)
4 (bedridden)	41 (19.7 %)
Living place before admission	
Home	151 (72.6 %)
Nursing and healthcare facility	54 (25.9 %)
Hospital	3 (1.4 %)
Infection site	
Pulmonary system	Subtotal = 45
Community-acquired pneumonia	7 (3.3 %)
Aspiration pneumonia	38 (18.2 %)
Urinary tract	Subtotal = 105
Pyelonephritis	20 (9.6 %)
Complicated pyelonephritis	67 (32.2 %)
Urosepsis	8 (3.8 %)
Prostatitis	5 (2.4 %)
Catheter related pyelonephritis	5 (2.4 %)
Skin & soft tissue	Subtotal = 58
Cellulitis	34 (16.3 %)
Severe cellulitis	18 (8.6 %)
MRSA suspected cellulitis	6 (2.8 %)
Previous antibiotic exposure within 48 h	29 (13.9 %)
Gram stain performed at ER	149 (71.6 %)
Blood culture positivity	34 (16.3 %)
Death	1 (0.4 %)

Table 2 shows a comparison of the initial antibiotics selected based on the Gram stain results or with the empirical antibiotics recommended by the guidelines. In the Gram stain group, narrow-spectrum antimicrobials were used most often, whereas in the guidelines group, broad-spectrum antimicrobials were selected most often.

**Table 2:** Initial antibiotics selected based on Gram stain results and on the Japanese guidelines

	Gram stain	Guidelines	*p* value
Narrow spectrum	*N* = 167	*N* = 44	0.0000*
Ampicillin	12	0	
Ampicillin/sulbactum	22	20	
Cefazolin	48	17	
Cefotiam	85	7	
Intermediate spectrum	*N* = 40	*N* = 77	0.0000*
Cefmeatazole	3	0	
Flomoxef	0	23	
Cefotaxime	10	2	
Ceftriaxone	18	24	
Ceftazidime	3	27	
Aztreonam	1	0	
Clindamycin	5	0	
Ciprofloxacin	0	1	
Broad spectrum	*N* = 10	*N* = 93	0.0000*
Piperacillin/tazobactum	0	27	
Cefepime	0	1	
Imipenem/cilastatin	3	9	
Meropenem	4	35	
Doripenem	0	8	
Biapenem	0	7	
Vancomycin	3	6	

*p < 0.05

Table 3 shows the culture results for each type of infection: pulmonary, urinary tract, and skin and soft tissue. The blood culture results are shown for skin and soft tissue infections, because useful specimens were not usually obtained. Seven of the *E. coli* isolates produced ESBLs, whereas no *K. pneumoniae* isolates produced ESBLs.

**Table 3:** Culture results for each pulmonary system, urinary tract, and skin and soft tissue infection

	*N* (%)
Sputum culture of pulmonary infection	Subtotal = 45
*Streptococcus pneumoniae*	11 (24.4 %)
*Haemophilus influenzae*	9 (20.0 %)
*Klebsiella pneumoniae*	6 (13.3 %)
*Moraxella catarrhalis*	5 (11.1 %)
*Pseudomonas aeruginosa*	4 (8.8 %)
*Staphylococcus aureus*	4 (8.8 %)
others	7 (15.5 %)
Urine culture of urinary tract infection	Subtotal = 105
*Escherichia coli*	76 (72.3 %)
*Klebsiella pneumoniae*	9 (8.5 %)
*Citrobacter koseri*	3 (2.8 %)
*Morganella morganii*	3 (2.8 %)
*Enterobacter aerogenes*	2 (1.9 %)
*Enterobacter cloacae*	2 (1.9 %)
*Enterococcus faecalis*	2 (1.9 %)
*Proteus mirabilis*	2 (1.9 %)
*Pseudomonas aeruginosa*	2 (1.9 %)
*Serratia marcescense*	2 (1.9 %)
Others	6 (5.7 %)
Blood culture of skin & soft tissue infection	Subtotal = 58
*Streptococcus dysgalactiae* subsp. *equisimilis*	9 (15.5 %)
*Steptococcus agalactiae*	1 (1.7 %)
negative	48 (82.7 %)

Table 4 shows the effectiveness of the initially selected antibiotics in each group. The antibiotics administered to both groups displayed high efficacy, approaching 90 %.

**Table 4:** Effectiveness of the initially selected antibiotics in each group

	Gram stain	Guidelines	*p* value
Effective	186 (89.4 %)	191 (91.8 %)	0.2071
Not effective	14 (6.7 %)	10 (4.8 %)	0.1999
Unknown	8 (3.8 %)	7 (3.3 %)	0.6865

Table 5 shows the antibiotic costs in each group. The total antimicrobial cost in the Gram stain group was less than half that in the guidelines group.

**Table 5:** Antimicrobial costs in each group

		Antibiotics total cost (yen)		
	*N*	Gram stain	Guidelines	Cost ratio
Pulmonary system	Subtotal = 45			
Community-acquired pneumonia	7	94,843	115,528	0.82
Aspiration pneumonia	38	635,846	1,940,352	0.33
Urinary tract	Subtotal = 105			
Pyelonephritis	20	447,282	764,816	0.58
Complicated pyelonephritis	67	2,021,718	4,559,744	0.44
Urosepsis	8	484,687	591,654	0.82
Prostatitis	5	100,652	142,056	0.71
Catheter related pyelonephritis	5	202,330	430,245	0.47
Skin & soft tissue	Subtotal = 58			
Cellulitis	34	555,997	1,279,216	0.43
Severe cellulitis	18	540,940	1,505,790	0.36
MRSA suspected cellulitis	6	324,756	1,565,758	0.21
Total	208	5,409,051	12,894,159	0.42

The actual number of days of intravenous antimicrobial administration during hospitalization for community-acquired pneumonia was 5.7 ± 1.3 (mean ± SD); for aspiration pneumonia, 6.2 ± 2.2; pyelonephritis, 9.7 ± 2.8; complicated pyelonephritis, 10.9 ± 2.3; urosepsis, 10.2 ± 4.3; prostatitis, 6.6 ± 5.1; catheter-related pyelonephritis, 12.0 ± 2.1; cellulitis, 8.0 ± 3.8; severe cellulitis, 10.8 ± 7.8; and MRSA-suspected cellulitis, 13.5 ± 9.3.

## Discussion

This study had three main findings. First, the antimicrobials selected based on the point-of-care Gram stains were significantly more often narrower-spectrum antibiotics than those selected based on the guidelines. Second, treatments based on the Gram stain and on the guidelines were equally highly effective. Third, the total antibiotic costs based on the Gram stain were less than half those based on the guidelines.

Antimicrobial resistance in common bacterial pathogens is a growing public-health concern worldwide [1], and appropriate antibiotic use is the most important modifiable factor when addressing this problem [17]. The Centers for Disease Control and Prevention (CDC) of the United States recently released *Core Elements of Hospital Antibiotic Stewardship Programs* [17]. This report states that facility-specific treatment recommendations should be based on national guidelines and local susceptibilities. In Japan, the Japanese Association for Infectious Diseases and the Japanese Society of Chemotherapy developed antimicrobial guidelines, the JAID/JSC Guide to the Clinical Management of Infectious Diseases 2011, which was updated in 2015 [18]. However, to date, no guidelines strongly recommend the use of the Gram stain as an essential antimicrobial stewardship tool.

In this study, we have demonstrated that Gram stains performed by in-house staff members contributed directly to the selection of significantly fewer broad-spectrum antibiotics than did the guidelines currently used in Japan. At Okinawa Chubu Hospital, all young trainee doctors have used Gram stains in the emergency room to select the most appropriate antibiotic for each patient for nearly 40 years [12]. They have been educated to practice Gram staining of sputum, urine, stool, pus, pleural effusion, ascites, and cerebrospinal fluid samples by themselves to detect infectious pathogens. This research demonstrates that point-of-care Gram staining frequently circumvents the use of broad-spectrum antimicrobials, especially in community-acquired or nursing-facility-associated infections.

The Gram stain is a basic technique, but its interpretation involves some skill. For example, the sensitivity and specificity of sputum Gram staining varies dramatically, depending on several factors [13, 19]. Therefore, it can be misleading and its use can be hazardous, especially if the interpreter is not well trained [19].

In our study, the Gram-stain-selected antimicrobials were as effective as the guideline-based ones, when the Gram stains were performed by in-house staff members. The results for the guidelines were derived from simulated calculations, and may therefore be inaccurate. However, nearly 90 % of the Gram-stain-based treatments were effective. The enrolled patients were mainly elderly, and 16.3 % of blood cultures were positive, but the case fatality rate was only 0.4 %. There are three reasons behind this surprising outcome.

First, the hospital’s Microbiology Laboratory reports the local antibiotic-resistance patterns every year. This information is useful when choosing an antimicrobial, as discussed in the Methods section. Healthcare facilities should have local antibiotic treatment guidelines that take local antibiotic resistance patterns into account [20].

Second, the Gram stain results were reviewed by well-trained senior residents or attending physicians to avoid their misinterpretation. After the culture results were returned, the trainee doctors confirmed their evaluation and considered the sensitivity and specificity of the Gram stains. Continual efforts such as these enhance their ability to interpret Gram stains. The microbiological culture results provide a guide to the appropriateness and duration of the antimicrobials and possible oral medications [21].

Third, even when the Gram-stain results recommended narrow-spectrum antimicrobials, staff members were trained to commence empirical broad-spectrum antimicrobials if the patient was in a critical condition or at risk of multidrug-resistant bacterial infection. Gram staining frequently identifies the etiological agent at the infection site, but the stained bacterial morphology cannot identify drug resistance.

At our hospital, the disadvantage of the Gram stain (possible misinterpretation) has been reduced through the continuous conscientious work of both staff members and attending physicians. The current generation of medical students and all doctors should receive education and ongoing training in antibiotic resistance and the prudent use of antimicrobials [21].

The implementation of the antimicrobial stewardship program has been associated with significant reductions in antimicrobial use and pharmacy costs [22]. The judicious use of antimicrobials has translated into improved patient outcomes and lowered health-care costs [23]. However, guidelines generally recommend that patients at risk of infection by resistant bacterial pathogens receive empirical broad-spectrum therapies. These increase the costs and possibly expose the patients to adverse events, such as *C. difficile*-associated disease [11].

This study has demonstrated that the calculated total drug cost based on Gram staining was less than half that based on the Japanese guidelines. This is because the narrow-spectrum antimicrobials are usually older drugs, and such classical drugs are cheaper than the latest broad-spectrum drugs. Therefore, Gram-stain-based treatments led not only to the better use of antibiotics, but also to more economical therapies.

Another advantage of Gram-stain-based therapies is that narrow-spectrum antibiotics limit the exposure of normal flora to antimicrobials. This reduces the *C. difficile*-associated disease rate [22] and the emergence of resistant pathogens [11]. In fact, carbapenem-resistant Enterobacteriaceae, such as *K. pneumoniae* carbapenemase (KPC)-producing bacteria, have not yet emerged in our hospital.

The development and spread of antibiotic resistance is multifactorial, and no single intervention can solve the problem [17]. Antimicrobial stewardship should include adaptable and customizable programs that can be designed to fit the infrastructure of any hospital [24]. However, the active use of Gram staining should be included in any antimicrobial stewardship program, because it has been demonstrated to be a practical, effective, and economical tool for primary-care physicians.

Our study had several limitations. First, the types of antimicrobials selected based on the guidelines were only simulated. We assumed that the prescribed antimicrobials were not de-escalated in the guidelines group after the culture results were received. This was because once a broader-spectrum antibiotic is commenced in Japan, it is not narrowed down in most cases, unless a specialist in infectious diseases intervenes. Therefore, the difference in the calculated drug costs for the Gram-stain-selected and guideline-selected therapies would be smaller than we calculated. Second, the guidelines have been designed for clinicians in Japan only, and are not available outside Japan. Therefore, the results cannot be directly applied to other countries. Third, the infection sites were limited to the pulmonary system, urinary tract, and skin and soft tissues, and diseases that require empirical broad-spectrum antimicrobials, such as meningitis, ascending cholangitis, endocarditis, and osteomyelitis, or critically ill patients, were not included. Our strategy is not applicable to these patients.

## Conclusions

The point-of-care Gram stain by in-house staff members dramatically reduced the use of broad-spectrum antimicrobials without affecting the effectiveness of treatment compared with that based on the Japanese guidelines. The drug costs were also less than half when the Gram strain was used. Therefore, Gram staining should be included in all antimicrobial stewardship programs.

## Abbreviations

ESBLs: Extended-spectrum of beta-lactamases
HIV: Human immunodeficiency virus
JAID: Japanese Association for Infectious Diseases
JSC: Japanese Society of Chemotherapy
KPC: *Klebsiella pneumoniae* carbapenemase
MRSA: Methicillin-resistant *Staphylococcus aureus*
